# Basal cell adenocarcinoma of the salivary gland: a morphological and immunohistochemical comparison with basal cell adenoma with and without capsular invasion

**DOI:** 10.1186/1746-1596-8-171

**Published:** 2013-10-21

**Authors:** Min Jung Jung, Jong-Lyel Roh, Seung-Ho Choi, Soon Yuhl Nam, Sang Yoon Kim, Sang-wook Lee, Kyung-Ja Cho

**Affiliations:** 1 Department of Pathology, University of Ulsan College of Medicine, Asan Medical Center, 388-1 Pungnap-dong, Songpa-gu, Seoul, 138-736 Korea; 2 Head and Neck Surgery, University of Ulsan College of Medicine, Asan Medical Center, Seoul, Korea; 3 Radiation Oncology, University of Ulsan College of Medicine, Asan Medical Center, Seoul, Korea

**Keywords:** Salivary gland neoplasm, Basal cell adenocarcinoma, Basal cell adenoma, β-catenin, Capsular invasion

## Abstract

**Background:**

It is often difficult to diagnose salivary gland tumors that exhibit basaloid features differentially. The aim of this study was to identify additional morphological and immunohistochemical characteristics that can aid the diagnosis of basal cell adenocarcinoma (BCAC) of the salivary gland.

**Methods and results:**

In total, 29 basal cell neoplasms [eight BCACs, 11 basal cell adenomas (BCAs) with capsular invasion, and 10 BCAs without capsular invasion] and 10 cases of adenoid cystic carcinomas (ACCs) were subjected to histopathology and immunohistochemical analyses for CK7, CK5/6, SMA, p63, calponin, p53, c-erbB2, CD117, β-catenin, EGFR, VEGF, Ki-67, and S100P protein expression. Compared to BCA without capsular invasion, the BCACs and BCAs with capsular invasion were more likely to be larger and have solid or cribriform patterns. Most BCACs and BCAs exhibited nuclear β-catenin expression. In all basal cell neoplasm cases, the clinical course after surgery with or without radiotherapy was indolent. β-catenin, CK5/6, CD117, and S100P protein were helpful for differentiating basal cell neoplasms from ACC.

**Conclusions:**

BCAs with capsular invasion shared several pathological features with BCACs, including a large size and frequent cribriform patterns but the malignant potential of these tumors seems highly limited and should be reexamined. β-catenin immunostaining may aid the differential diagnosis between basal cell neoplasms and ACCs.

**Virtual slides:**

The virtual slide(s) for this article can be found here: http://www.diagnosticpathology.diagnomx.eu/vs/9637819101988153

## Introduction

Basal cell adenocarcinoma (BCAC) is a rare malignant tumor of the salivary gland that was included in the 1991 World Health Organization (WHO) classification. To date, several studies and case reports that describe this tumor have been published [1-13]. Despite this, a solid consensus regarding the features that can be used to distinguish BCAC from basal cell adenoma (BCA) has not been obtained. In 2005, the WHO classification simply defined BCAC as being an infiltrative epithelial neoplasm that is similar to BCA [14].

While BCAC can be distinguished from BCA on the basis of the infiltration of tumor cells into the parotid parenchyma, dermis, skeletal muscle, or periglandular fat [14], this infiltration is not severe because BCAC is essentially a low-grade malignancy. Thus, it can be difficult to distinguish early stage BCACs from BCAs; it is also difficult to distinguish BCACs from BCAs that exhibit minimal capsular invasion. The diagnosis of BCACs is also hampered by the fact that they can have a solid or cribriform pattern that mimics the features of adenoid cystic carcinoma (ACC). Although three studies have searched for immunohistochemical markers that can be useful for diagnosing BCAC, meaningful markers have not been found [3,8,12]. Thus, the present study sought to identify the clinical, histopathological, and immunohistochemical characteristics of BCAC that allow it to be differentiated from BCA and ACC.

## Methods

### Case selection

Two pathologists reviewed the surgical pathology files of the Asan Medical Center for primary basaloid salivary gland tumors that were diagnosed between 1993 and 2010. Tumors were only selected for analysis if they had unequivocal basal cell features and an invasive growth pattern at the periphery of the tumor, regardless of its extent. We retrieved 19 cases. These 19 tumors had initially been diagnosed as BCAC (n=13), BCA (n=2), and ACC (n=4). However, re-analysis of these cases revealed that only eight met the histological requirements of BCAC. The remaining 11 cases were classified on the basis of current diagnostic criteria as BCA with capsular invasion, although there remained some suspicion that some of these tumors could be BCAC because of the presence of capsular invasion. These 19 cases included two cases that had been referred from outside hospitals. Another 10 cases each of typical BCA without capsular invasion and ACC that were diagnosed from 2009 to 2011 were also selected by the pathologists to serve as comparators in the tissue microarray and immunohistochemical analyses. The clinical data of all 39 cases were obtained by reviewing the medical records.

### Histopathologic examination

During the review of the slides, the two pathologists analyzed the architectural patterns (tubular, trabecular, solid, cribriform, or membranous, mixed), mitotic counts, lymphovascular or perineural invasion, and other notable changes in the basal cell neoplasms (BCNs).

### Immunohistochemical analysis

Formalin-fixed paraffin-embedded blocks were available for 17 of the 19 BCAC and BCA with capsular invasion cases (seven BCACs and 10 BCAs with capsular invasion), the 10 BCA without capsular invasion cases, and the 10 ACC cases. These blocks were not available for the two referred cases. Tissue microarrays were generated from the blocks by using a manual tissue arrayer (Pathology Devices, Westminster, MD, USA). Thus, three 1.5 mm cores were taken from the donor blocks and arrayed into recipient blocks. Thereafter, 4 μm tissue microarray sections were subjected to immunoperoxidase staining by using a Ventana autostainer and an ultra view DAB detection kit (Ventana, Tucson, AZ, USA) according to the manufacturer’s instructions. The primary antibodies used in this study are listed in Table 1. Two observers then analyzed the immunohistochemical reactivity to determine the staining patterns and intensity, and which cell types were stained. The cytoplasmic immunostaining of cytokeratin 7 (CK7), cytokeratin 5/6 (CK5/6), smooth muscle actin (SMA), calponin, and vascular endothelial growth factor (VEGF), the membranous/cytoplasmic staining of CD117, the nuclear staining of p63, p53, and S100P protein, and the nuclear or cytoplasmic/membranous staining of β-catenin were graded as follows: negative (no staining or < 5% positive cells), focally positive (5–30% positive cells), and diffusely positive (≥30% positive cells). EGFR and c-erbB2 expression were evaluated on the basis of the membrane staining pattern and intensity, as follows: negative (no staining, 0), weak (partial or weak complete membrane staining, 1+), moderate (moderate membrane staining, 2+), and strong (strong membrane staining, 3+). A sample was deemed to be positive for EGFR or c-erbB2 expression if it showed moderate or strong membrane staining. The nuclear and cytoplasmic/membranous staining for β-catenin was assessed separately. Ki-67 expression was evaluated by calculating the Ki-67 labeling index.

**Table 1:** The primary antibodies used in this study

**Primary antibodies against**	**Source**	**Dilution**	**Clone**
Cytokeratin 7	DAKO	1:400	Mouse monoclonal
Cytokeratin 5/6	ZYMED	1:200	Mouse monoclonal
SMA	DAKO	1:400	Mouse monoclonal
p63	NOVO	1:25	Mouse monoclonal
calponin	NEOMARKERS	1:3000	Mouse monoclonal
p53	DAKO	1:3000	Mouse monoclonal
c-erbB2	DAKO	1:500	Mouse monoclonal
CD117	DAKO	1:400	Rabbit polyclonal
β-catenin	ZYMED	1:2000	Mouse monoclonal
EGFR	ZYMED	1:100	Mouse monoclonal
VEGF	PHARMINGEN	1:500	Mouse monoclonal
Ki-67	ZYMED	1:100	Mouse monoclonal
S100P protein	Protein tech	1:100	Rabbit polyclonal

EGFR, epidermal growth factor receptor; SMA, smooth muscle actin; VEGF, vascular endothelial growth factor.

### Statistics

To compare the different tumor types in terms of their characteristics, *t*-tests were used with quantitative variables and Fisher exact tests were used with categorical variables. A *P*-value of < 0.05 was considered to indicate statistical significance. Statistical analysis was performed by using SPSS version 18.

## Results

### Clinical characteristics

As shown in Table 2, six and two of the eight patients with BCAC were female and male, respectively. This group did not differ significantly from the BCA with and without capsular invasion groups in terms of gender distribution, although there was a notable female predominance in all BCN groups. The BCAC group was on average 60.6 years old (range, 51–79 years), which meant that this group was on average more than a decade older than the patients with BCA with capsular invasion (47.9 years) and the patients with BCA without capsular invasion (47.5 years). These differences between the BCAC cases and the other two groups were statistically significant (*P* < 0.05). All BCACs were located in the parotid gland, showed a predilection for the left side (6:2). The BCAC group did not differ significantly from the BCA with and without capsular invasion groups in terms of site. The BCACs were 3.5 cm in diameter on average (range, 1.6–5.0 cm), while the BCAs with and without capsular invasion were on average 3.1 and 1.9 cm, respectively. The BCACs were significantly bigger than the BCAs without capsular invasion (*P* < 0.001) but the difference between BCACs and BCAs with capsular invasion did not achieve statistical significance.

**Table 2:** Clinical characteristics of patients with basal cell neoplasms

		**BCACs**	**BCAs with ci.**	**BCAs without ci.**
		**(n=8)**	**(n=11)**	**(n=10)**
Age (yrs)	Mean (range)	61 (51–79)	48 (27–67)	48 (27–64)
Sex	Female/Male	6/2	8/3	9/1
Tumor size (cm)	Mean (range)	3.5 (1.6–5.0)	3.1 (1.8–7.5)	1.9 (1.3–2.5)
Site	Parotid gland (Lt/Rt)	8 (6/2)	11 (9/2)	9 (5/4)
	Submandibular gland	0	0	1
Treatment	Surgery	3	4	10
	Surgery + RT	5	7	0
Follow-up	Recurrence	0	0	0
	NED	6	7	10
	Loss	2	4	0

BCAC, basal cell adenocarcinoma; BCA, basal cell adenoma; ci., capsular invasion; Lt, left; NED, no evidence of disease; Rt, right; RT, radiotherapy.

All 29 patients with BCNs were initially treated by surgery (Table 2). Twelve of the nineteen cases of BCAC and BCA with capsular invasion (five BCACs and seven BCAs with capsular invasion) also received postoperative radiotherapy. None of the BCAs without capsular invasion received radiotherapy. None of the 19 patients with BCAC or BCA with capsular invasion developed local recurrences or distant metastases, and 18 have lived with no evidence of disease for 27 to 233 months (mean, 90 months). The remaining patient had been diagnosed with BCAC and died of subarachnoid hemorrhage from a ruptured aneurysm 2 years after diagnosis. None of the patients with BCA without capsular invasion died or had recurrence.

### Microscopic analyses

**Figure 1:**
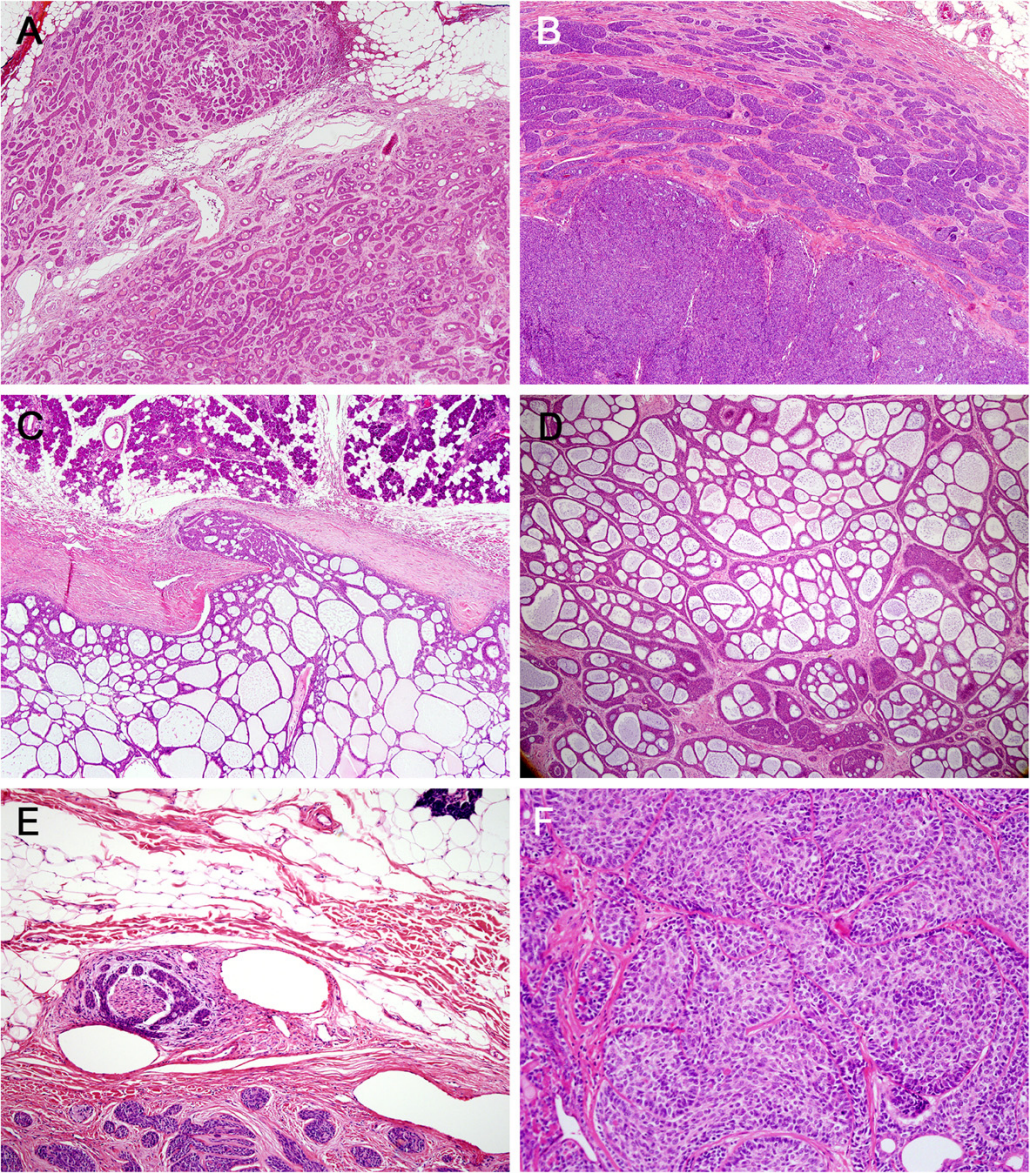
**Histologic findings of basal cell adenocarcinoma and basal cell adenoma with capsular invasion. A**. A basal cell adenocarcinoma that is unencapsulated and is invading into the adjacent fat. **B**. A basal cell adenoma with capsular invasion. Variably-sized solid nests are streaming from the solid component and are attenuating parts of the capsule. **C**. The cribriform variant of the basal cell adenoma with capsular invasion shows a focal tongue-like projection into the capsule. **D**. The cribriform pattern of the basal cell adenocarcinoma with capsular invasion mimics adenoid cystic carcinoma in that it presents with tumor islands with multiple holes. However, it does not show accompanying invasive nests with true lumina, which are seen in adenoid cystic carcinoma. **E**. A basal cell adenocarcinoma that exhibits invasive growth with associated perineural invasion. **F**. The solid form of basal cell adenocarcinoma. The tumor is composed of basaloid cells, which occur concomitantly with vague two-cell morphologies and some palisading at the periphery.

Microscopic analysis revealed that the BCACs were mostly encapsulated and had invasive areas at the periphery that varied in extent but were mostly minimal (Figure 1A). The BCAs with capsular invasion showed a common pattern of invasion: small solid nests of tumor cells that were streaming from the main mass caused attenuation of moderate parts of the capsule (Figure 1B). Another minimal capsular invasion pattern was characterized by focal destruction of the capsule by tongue-like projections or tumor cell buds that are continuous with the main mass, while most of the capsule remains well-preserved (Figure 1C).

The BCACs exhibited a mixture of growth patterns, including tubular, trabecular, solid, membranous, and cribriform (Table 3). The predominant architectural growth pattern was solid (62.5%), followed by cribriform (25%). Similarly, the predominant patterns in BCAs with capsular invasion were solid and cribriform (these two patterns accounted for 72.7% of these tumors). By contrast, in BCAs without capsular invasion, the trabecular pattern predominated (60%) and the cribriform pattern was not observed at all. The cribriform pattern in the BCACs or BCAs with capsular invasion was characterized by large nests, large expansile lumens, and thin interluminal walls, and consisted of a homogenous cell type (Figure 1D).

**Table 3:** Pathological characteristics of basal cell neoplasms

		**BCACs**	**BCAs with ci.**	**BCAs without ci.**
		**(n=8)**	**(n=11)**	**(n=10)**
Predominant pattern	Solid	5 (62.5%)	4 (36.4%)	3 (30.0%)
	Cribriform	2 (25.0%)	4 (36.4%)	0 (0.0%)
	Trabecular	0 (0.0%)	3 (27.3%)	6 (60.0%)
	Tubular	1 (12.5%)	0 (0.0%)	1 (10.0%)
Mitosis	> 4/10HPFs	1 (12.5%)	2 (18.2%)	0 (0.0%)
Perineural invasion		1 (12.5%)	0 (0.0%)	0 (0.0%)
Lymphovascular invasion		0 (0.0%)	0 (0.0%)	0 (0.0%)
Presence of daughter mass		3 (37.5%)	1 (9.1%)	0 (0.0%)
Cystic change		3 (37.5%)	3 (27.3%)	0 (0.0%)
Squamous differentiation		1 (12.5%)	1 (9.1%)	0 (0.0%)

BCAC, basal cell adenocarcinoma; BCA, basal cell adenoma; ci., capsular invasion; HPF, high power field.

In terms of mitotic count, one of the eight BCACs and two of the eleven BCAs with capsular invasion exhibited more than four mitoses per ten high power fields (Table 3). However, the mitotic count of these tumors was generally low and none of these tumors showed necrosis. Only one case of BCAC showed perineural invasion (Figure 1E).

At the cytomorphological level, BCAC could not be distinguished from BCA with or without capsular invasion. These tumors consisted of relatively isomorphic basaloid cells with little cytoplasm and elongated hyperchromatic nuclei. The cells often displayed vague two-cell morphologies, especially in tubulotrabecular types. The peripherally located cells tended to have more basophilic nuclei and scant cytoplasms while the inner cells within the nests, tubules, and cords tended to be more pale and plump. This contrast was reminiscent of epithelial-myoepithelial structures, but the inner cells maintained the basaloid features (thus differing from the eosinophilic tubular cells of ACC) and the outer cells rarely exhibited clear cytoplasms. The tumor cells in the solid cases were uniform with some palisading at the periphery (Figure 1F).

### Immunohistochemical analysis

**Figure 2:**
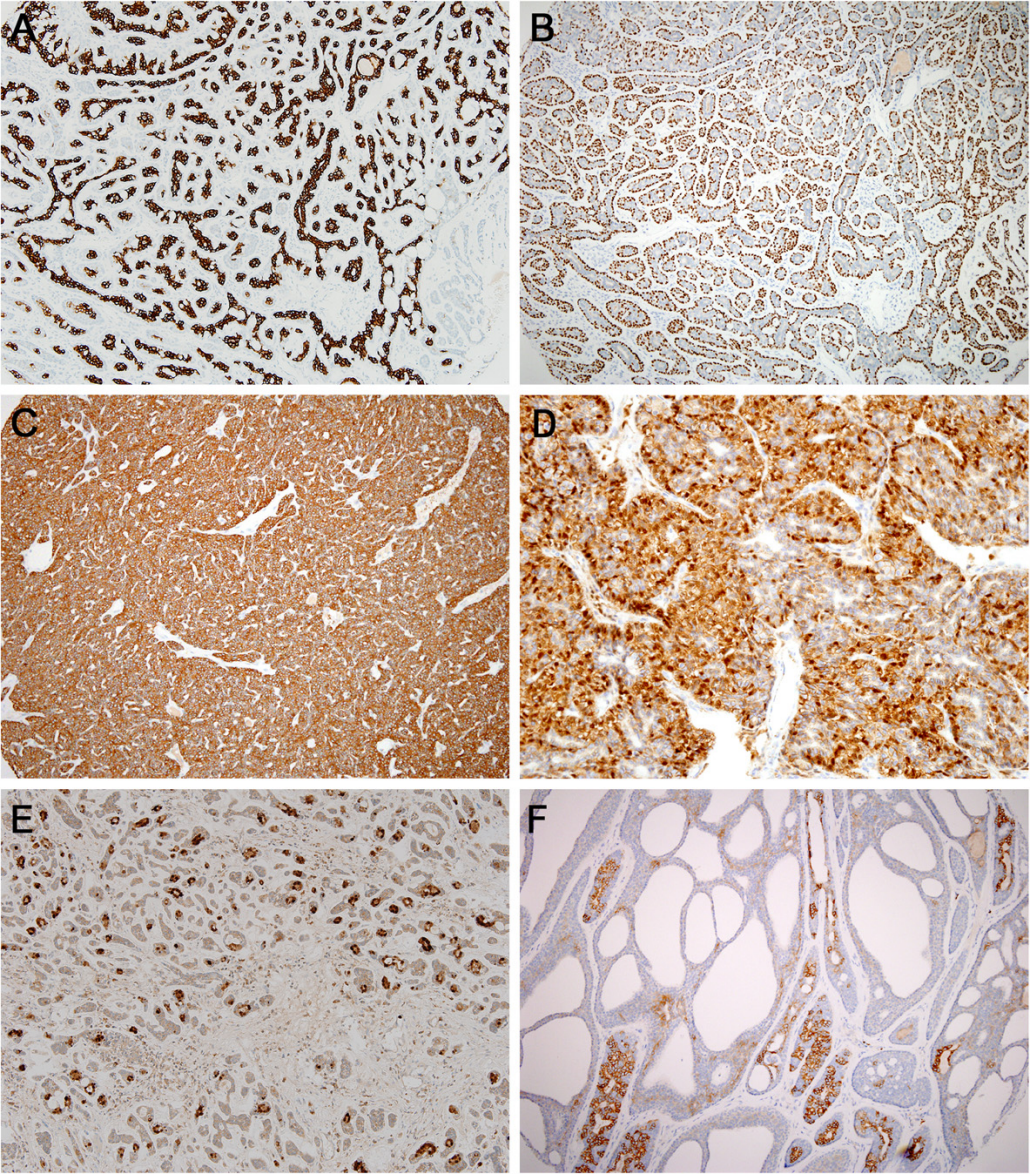
**Immunohistochemical findings of basal cell neoplasm and adenoid cystic carcinoma. A & B**. Immunostaining of basal cell neoplasms for CK7 **(A)** and p63 **(B)** illustrate the pattern of epithelial-myoepithelial differentiation, especially in tubular or trabecular type tumors. **C**. CK5/6 immunostaining reveals that most basal cell adenocarcinomas exhibit diffuse cytoplasmic staining, which differs from the staining pattern in adenoid cystic carcinoma. **D**. Nuclear β-catenin expression in a basal cell adenocarcinoma. **E**. Unlike basal cell adenocarcinomas, which are negative for S100P protein, most adenoid cystic carcinomas show nuclear expression of S100P protein. **F**. Unlike adenoid cystic carcinoma, which is diffusely and strongly positive for CD117, basal cell adenocarcinoma shows focally and weakly to moderately positive CD117 staining.

All 27 BCNs that were subjected to immunohistochemical analysis expressed CK7, SMA, p63, and calponin. The expression pattern of these epithelial or myoepithelial/basal cell markers mimicked epithelial-myoepithelial differentiation. The inner/luminal cells tended to express CK7 (Figure 2A) while the outer/peripheral cells expressed SMA and p63 (Figure 2B). The expression pattern of calponin was similar to that of SMA and p63 but the staining was less intense and extensive.

All BCAC cell types were positive for CK5/6, which was not observed for SMA, p63, and calponin (Figure 2C). In addition, with the exception of one BCA with capsular invasion that showed focal positivity for CK5/6, all 26 remaining BCNs expressed CK5/6 with a diffuse pattern (Table 4). Some cases expressed CK5/6 more strongly in peripherally located cells. Although all 10 ACCs also expressed CK5/6, the positivity was mostly focal, and mainly in the inner epithelial cells. This is interesting because CK5/6 is a myoepithelial/basal marker. The BCNs not only differed from the ACCs in terms of CK5/6 expression, they also differed in nuclear β-catenin and S100P protein expression. Thus, nuclear β-catenin was expressed by 70–100% of the BCNs (Figure 2D) and 0% of the ACCs, and S100P protein was expressed by 0–10% of the BCNs but by 50% of the 10 ACCs (Figure 2E). With regard to CD117, 57%, 60%, 100%, and 100% of the BCAC, BCA with capsular invasion, BCA without capsular invasion, and ACC cases expressed this marker, respectively (Figure 2F). However, this marker exhibited focal CD117 expression in most BCNs whereas all ACCs diffusely expressed CD117. In terms of p53, while only 3 of the 27 BCNs (11%) were focally positive for this marker, 6 of 10 ACCs (60%) were positive for it. Moreover, in all ACCs, the Ki-67 labeling index exceeded 5% whereas most BCNs had a low ki-67 labeling index (< 5%). All BCNs and ACCs expressed VEGF, while none expressed c-erbB2.

**Table 4:** Immunohistochemical characteristics of basal cell neoplasms and adenoid cystic carcinomas

	**BCACs (n=7)**	**BCAs with ci. (n=10)**	**BCAs without ci. (n=10)**	**ACCs (n=10)**
Cytokeratin 5/6*	7 (100%)	9 (90%)	10 (100%)	3 (30%)
S100P protein	0 (0%)	1 (10%)	1 (10%)	5 (50%)
β-catenin**	7 (100%)	7 (70%)	10 (100%)	0 (0%)
CD117	4 (57%)	6 (60%)	10 (100%)	10 (100%)
EGFR	3 (43%)	2 (20%)	9 (90%)	5 (50%)
p53	0 (0%)	1 (10%)	2 (20%)	6 (60%)
Ki-67^$^	0 (0%)	2 (20%)	2 (20%)	10 (100%)

*diffuse staining only; **nuclear staining only; ^$^Ki-67 labeling index > 5%

ACC, adenoid cystic carcinoma; BCAC, basal cell adenocarcinoma; BCA, basal cell adenoma; ci., capsular invasion; EGFR, epidermal growth factor receptor.

## Discussion

Due to the low incidence of and insufficient information on BCAC, it is often difficult to diagnose this tumor. The WHO classification indicates that while BCAC is cytologically and histomorphologically similar to BCA, it is distinguished by the fact that it is also an infiltrative epithelial neoplasm that has a potential for metastasis. However, considering that it has now been officially accepted that pleomorphic adenoma has infiltrative and metastatic potential, basal cell adenoma may also be considered as an infiltrative neoplasm and the category of BCAC can be questioned. Moreover, the current diagnostic criteria that are used to diagnose BCAC can be impracticable because BCAC with extensive infiltrative growth, such as into skeletal muscle or skin, is rarely encountered.

In the present study, the infiltrative potential of all tumors was regarded as being meaningful. Consequently, all BCNs that showed invasion into the periphery, however focal it might have been, were considered to be BCAC candidates. The morphological, immunohistochemical, and clinical differences of the BCNs according to the extent of invasion were then determined. With regard to malignant potential, this could not be judged in the cases with capsular or extracapsular invasion because none of our patients manifested recurrences or metastases after surgery with or without radiotherapy. However, the biological aggressiveness of BCAC needs to be reexamined, because while regional recurrences or distant metastases have been described since BCAC was first reported [1,2,4,9-12], some authors have noted that certain cases with recurrences or metastases have unusual histological features that are seen in other similar tumors, such as ACC [9,12]. In addition, most studies acknowledge that BCAC is a very low-grade tumor [4,5]. One exception, the study by Nagao *et al*., [10] found that BCAC had solid morphology, a high Ki-67 labeling index, p53 overexpression, and poor behavior. The disparities between these tumors and those in our and other studies cast doubt on the true identity of the tumors of Nagao *et al*.

In the present study, the BCACs and BCAs with capsular invasion were on average larger (3.5 and 3.1 cm, respectively, with the largest tumor being 7.5 cm) than the BCAs without capsular invasion (1.9 cm). However, these groups did not exhibit any cytomorphological differences. The only notable morphological dissimilarity was that the BCACs and BCAs with capsular invasion frequently had cribriform or solid growth patterns, unlike the BCAs without capsular invasion. However, the cribriform or solid patterns of BCAC can be misleading, resulting in the misdiagnosis of ACC, which is much more aggressive than BCAC. When the cribriform structures in BCACs were examined carefully, they were found to differ from the cribriform structures in ACC, as they had large expansile lumens, thin interluminal walls, bland and uniform cells with elongated small nuclei, and lacked definite two-cell populations. Moreover, the solid structures in BCACs were tightly apposed, often with jigsaw puzzle pattern or peripheral palisading. By contrast, solid variant ACC usually showed high-grade cytological features, including marked nuclear atypia and frequent mitoses.

To characterize BCAC immunohistochemically in the present study, 13 antibodies against various markers were used. While the BCACs, BCAs with capsular invasion, and BCAs without capsular invasion did not have clearly different profiles, S100P, β-catenin, CD117, and CK5/6 staining may help to make a differential diagnosis between BCNs and ACC. S100P protein has been proposed to be an initiator of carcinogenesis and is reported to play an important role in the malignant transformation of ductal cells of pleomorphic adenoma [15,16]. Our analysis of S100P expression in BCNs and ACCs revealed that most BCNs did not express this protein but 50% of ACCs showed nuclear S100P protein positivity, albeit with varying extents. Thus, S100P may be a useful marker for distinguishing ACCs from BCNs.

Various malignancies exhibit the nuclear expression of β-catenin. Several studies have also shown that ACC and other salivary gland tumors, such as epithelial-myoepithelial carcinoma, pleomorphic adenoma, BCA, and BCAC, express β-catenin [17-20]. In particular, two studies showed that BCA has nuclear β-catenin expression [17,18]. One of these, by Kawahara *et al*., concluded that while nuclear β-catenin expression may be a helpful marker for diagnosing BCA, it is not useful in the differential diagnosis between BCA and BCAC. They also showed that 154 other salivary gland tumors, including ACC, do not have nuclear β-catenin expression [18]. By contrast, two other studies suggest that some ACCs do express nuclear β-catenin [21,22]. Nevertheless, our results concur with the findings of Kawahara *et al*. [18]: all ACCs were negative for nuclear β-catenin staining, and all BCACs and BCAs without capsular invasion, and most BCAs with capsular invasion (7/10), showed nuclear positivity for β-catenin immunostaining. Interestingly, the three BCAs with capsular invasion that did not show nuclear β-catenin staining had higher mitotic activity (>10/10 HPFs) or apoptosis. Notably, most BCNs and ACCs also showed cytoplasmic/membranous positivity for β-catenin. It is generally well known that activation of Wnt signaling increases the cytoplasmic levels of β-catenin, resulting in increased nuclear β-catenin levels. A recent study of 45 salivary gland tumors revealed that various benign and malignant salivary gland tumors had cytoplasmic/membranous β-catenin expression [20]. A mutation in CTNNB1 (the gene that encodes β-catenin) may explain why BCAs show nuclear β-catenin expression [18,22]. Thus, although the significance of the nuclear expression of β-catenin in BCNs is currently not clear, it may be useful for differentially diagnosing between BCNs and ACCs.

CD117 is a recently recognized marker of ACC [23,24]. Surprisingly, however, many BCNs showed membranous/cytoplasmic CD117 positivity, some with weak to moderate intensity. When Edwards *et al*. compared ACC and monomorphic adenoma in terms of CD117 expression, they concluded that it is not a useful marker for differential diagnosis [25]. In the present study, however, the staining intensity of CD117 expression was stronger in ACCs than in BCNs, mainly in the luminal epithelial cells. Thus, this marker may still be useful for the diagnosis of ACC.

The present study showed that differential diagnosis between BCA, BCAC, and ACC was unlikely to be aided by the various epithelial and myoepithelial/basal cell markers that were tested. However, unlike the other markers, CK5/6 immunostaining indicated a difference between BCNs and ACCs. The antibody mainly stained the inner luminal cells of ACCs, whereas BCNs showed a diffuse staining pattern. In the past, BCAs were considered to be “monomorphic and isocellular”, but it was shown recently that they do not really lack myoepithelial differentiation [13,26]. The results of the present study with selected epithelial and myoepithelial markers were not inconsistent with these arguments. However, the diffuse expression of CK5/6 and lack of calponin expression in BCAs and BCACs may reflect the isomorphic basal cell character of these tumors.

Analysis of the Ki-67 labeling index and p53 expression rate in the present study revealed both were higher in ACCs than BCNs: while only 4 of 27 BCNs had a Ki-67 index > 5%, all ACCs had a Ki-67 index of approximately 20%. The BCACs also showed low p53 expression (< 5%), which indicates the low-grade nature of this tumor.

Recently, Soave *et al*. studied expression of CD44 and CD24 in malignant salivary gland tumors, and demonstrated their relationship with tumor site and stage, but did not provide differential diagnostic utility of CD44 and CD24 [27]. They described weak membranous CD44 positivity in 2 of 5 BCAC.

## Conclusions

BCNs with an invasive periphery and definable BCACs shared pathological features such as a large size, frequent cribriform patterns, and the expression of various proteins. However, BCNs without capsular penetration remained difficult to diagnose, not only because of the lack of invasive potential, but also because of the lack of immunohistochemical or molecular evidence supporting the notion that BCAs with capsular invasion may be early BCACs. Given the clinical outcome of the 19 tumors that had extracapsular or capsular invasion and were larger than the BCAs without capsular invasion, it can be concluded that BCAC is a low-grade tumor with little propensity for metastasis or recurrence. This raises doubt regarding the malignant potency of this tumor.

## Competing interests

The authors declare that they have no competing interests.

## Authors’ contributions

KC designed the study, analysed histological and immunohistochemical slides and wrote the manuscript. MJJ performed the statistical analysis, participated in histological and immunohistochemical evaluation and wrote the manuscript. SC, SYN, SYK and SL collected the patients’ clinical information and obtained the follow-up data. All authors have read and approved the final manuscript.
